# Backstep scanning ion conductance microscopy as a tool for long term investigation of single living cells

**DOI:** 10.1186/1477-3155-7-7

**Published:** 2009-10-27

**Authors:** Patrick Happel, Irmgard D Dietzel

**Affiliations:** 1 Department of Molecular Neurobiochemistry, Ruhr-University Bochum, D-44870 Bochum, Germany; 2 Central Unit for Ion Beams and Radionuclides (RUBION), Ruhr-University Bochum, D-44870 Bochum, Germany

## Abstract

Scanning ion conductance microscopy (SICM) is a suitable tool for imaging surfaces of living cells in a contact-free manner. We have shown previously that SICM in backstep mode allows one to trace the outlines of entire cell somata and to detect changes in cellular shape and volume. Here we report that SICM can be employed to quantitatively observe cellular structures such as cell processes of living cells as well as cell somata of motile cells in the range of hours.

## Findings

In order to obtain quantitative information about the dynamics of topographic changes as occur during cell migration, long term recordings of living cells are required. While the trajectories of cells can be followed with light microscopy, more complex topographic details of changes in shape can be obtained using scanning probe techniques. For example, atomic force microscopy (AFM) [1] has succesfully been used to observe patches of the cell membrane of living monkey kidney cells for hours as well as to reveal changes and detailed information about the structure of the growth cone of living hippocampal neurons [2-4]. Such applications require repeated scanning over the range of hours. Using this technique, however, the small physical force exerted leads to a visualization of the cytoskeleton rather than the membrane contours when imaging the cell somata [3]. Furthermore, repeated scanning of the membrane may lead to damage or contamination of the probe due to the adhesive forces between tip and glycoproteins [5].

SICM is essentially a contact-free scanning technique which uses electrical resistance changes to detect the distance between the scanning tip and an insulator [6]. The first successful scans of living cell surfaces with this method have been reported on cultured melanocytes and human colon cancer cells using a constant distance operating mode [7]. The observation of microvillar dynamics over the range of ten minutes [8] as well as recordings of cell somata within a confluent cell layer over 24 h [9] have been demonstrated using SICM. In order to obtain stable recordings from steep and overhanging membranes of non-confluent, single cultured neural cells we have introduced the pulse-mode SICM with floating backstep operation mode to image the topography of single cell bodies of neural cells and thus to monitor single cell shape and volume [10-12]. Recent investigations confirmed the necessity to operate SICM in backstep-type modes if images from entire cells with steep slopes are required and showed that the technique can be extended to a lateral resolution of 50 nm [13]. However, the actual resolution of SICM images is still a matter of debate [14]. Backstep SICM has also been applied recently to investigate and guide growth cones of leech neurons [15]. To our knowledge the application of SICM to obtain long term recordings of moving entire living cell somata in the range of hours has not yet been shown. Here we show examples of the application of backstep SICM to investigate changes in the shape of a terminal part of a nerve cell process and of two contact-forming cells as well as of recordings of the trajectories of a moving oligodendrocyte precursor cell (OPC) for several hours.

### Preparation and cell culture

Neural cells were obtained and cultured as described in [16] (protocol for mixed cultures) with the exception of using a cell density of 50 × 10^3^-100 × 10^3^ cells per cm^2^. Oligodendrocyte precursor cells were obtained and cultured as previously described in [17] but instead of changing the medium to proliferation/differentiation medium cells were continuously kept in a mixture of DMEM/Ham's F12 (1:1) supplemented with 10% fetal calf serum.

### SICM measurements

SICM measurements were performed using the pulse-mode SICM previously described [10] operating in floating backstep mode [11]. Briefly summarized, current pulses of predefined height were applied and the required voltage measured. The pulse height measured far away from the sample surface was used to define the basal resistance. Then, the probe was approached towards an insulating surface until the resistance exceeded a predefined threshold. A resistance increase of 3% with respect to the basal resistance was used in the present experiments to stop the approach. The *x*, *y*, *z*-coordinates of this point were stored for later reconstruction of the sample surface topography from successively measured points of equal resistance changes. To reduce scanning time the distance that the probe was dragged back was adjusted corresponding to the differences in height detected in a preceeding low resolution prescan. Scanning probes, filled with bath saline (containing in mM: NaCl 110, KCl 5.4, CaCl_2 _1.8, MgCl_2 _0.8, Glucose 10, HEPES 10), had an inner diameter of about 1 μm and an access resistance of about 4 MΩ. The scans were performed in 3.5 cm plastic petri dishes coated with poly-L-Lysin in Leibovitz-15 medium. Data were processed using Matlab and ImageJ software.

### Long term observation of the terminal part of a nerve cell process

**Figure 1:**
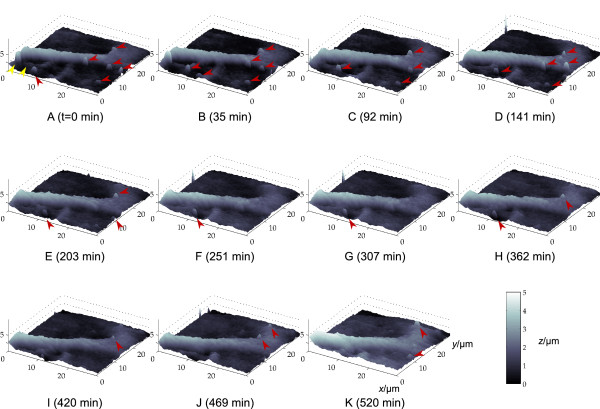
**Long term observation of the terminal part of a nerve cell process**. Eleven successive scans of the terminal part of a nerve cell process performed within 520 minutes. Axes scales represent micrometers as denoted in (K). Yellow arrows in (A) indicate a structure that gradually merges with the neurite. The lamellipodium is clearly visible on the right hand side of each image as well as various filopodia sticking out of the neurite (marked by the red arrows). Scanning step sizes were 500 nm and 100 nm in lateral and vertical direction, respectively.

**Figure 2:**
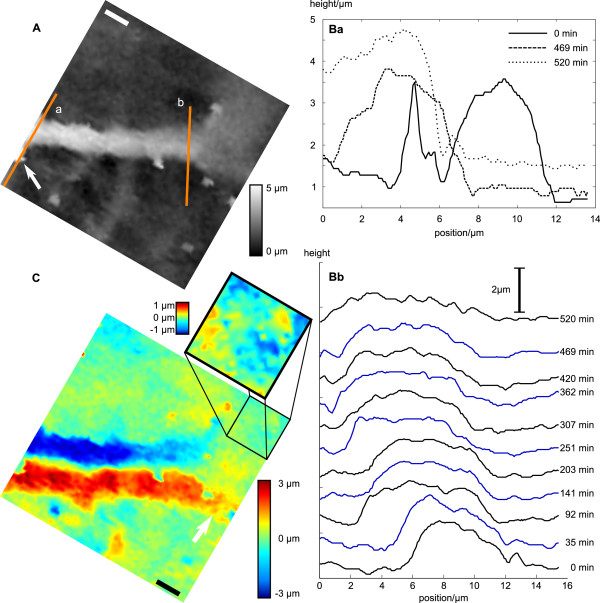
**Analysis of the topographical changes of the neurite shown in Fig. 1**. (A) Top view of the first scan of the terminal part of the nerve cell process (see Fig. 1 A). Orange lines indicate the positions of the height profiles shown in (B). (C) Difference image between frame A and J from Fig. 1. Lateral scale bars in (A) and (C) represent 3 μm. The color bar on the right indicates calibration of the *z*-axis in the large image, small color scale corresponds to the inset. Red and blue areas denote in- and decreases of height with time, respectively. Inset shows magnified lamellipodium with increased contrast.

The terminal part of a nerve cell process of a rat hippocampal neuron was imaged eleven times in 520 minutes [see Additional File 1]. Images are shown unfiltered but interpolated by cubic splines in Figure 1. Images were obtained with a lateral step size of 500 nm and a vertical step size of 100 nm, acquisition time was about 20 minutes per frame. In the course of the scanning period the neurite moved towards *y *= 0 μm merging with the structure marked by the yellow arrows in Fig. 1A. The lamellipodium is visible as the delta-formed structure at the end of the neurite (right hand side of the images). Various membrane changes are visible due to appearing and disappearing filopodia (marked by the red arrows in Fig. 1) the height of which appeared from 150 nm to 300 nm (see [14] for the restrictions of height detection via SICM of small objects). Figure 2 shows the topographic changes over time in more detail. Fig. 2Ba depicts the height profile along the orange line drawn in Fig. 2A (marked with a) from the frames A (solid line, 0 min), J (dashed line, 469 min) and K (dotted line, 520 min) from Fig. 1. Since overall height increased in scan K the height profiles of frames A and J are easier to compare. The position of the neurite shifted towards negative *y*-direction whereas its width nearly remained constant (about 6 μm). The process located left of the neurite in A (marked by the white arrow in Fig. 2A) gradually merged with the neurite, completely disappeared in frame F (Fig. 1) and thus is undetectable in the height profiles of scans J and K.

The height profile detected along the orange line marked as b in Fig. 2A is depicted in Fig. 2Bb. Profiles corresponding to increasing scanning time are displayed in the successive traces from bottom to top. In contrast to the profiles depicted in Fig. 2Ba the neurite only shifted slightly towards negative *y*-direction at this position. On the other hand it widened (from about 6 μm to about 9 μm) and flattened a bit, which could have been due to a slight retraction of the lamellipodium. This becomes visible in the difference image shown in Fig. 2C which has been created from the subtraction of frame A from frame J (Fig. 1). Here green areas denote areas without any changes in height, red areas correspond to areas of increasing height and blue areas represent areas of decreasing height. Whereas the position of the neurite had clearly shifted towards the negative *y*-direction (large parallel dark blue and dark red areas) the position of the lamellipodium changed only slightly. The slight retraction is visible in the inset in which contrast has been increased (indicated by the small color scale bar shown in the upper left of Fig. 2C) and by the light red and orange area indicated by the white arrow. This indicates that the observed movement occurred not due to drifts in the image frame that would either result in a similar shift of the lamellipodium or, if the image frame drift was circular, result in an angular instead of a parallel dislocation of the neurite.

### Long term observation of two neural cells establishing a new cell-cell contact

**Figure 3:**
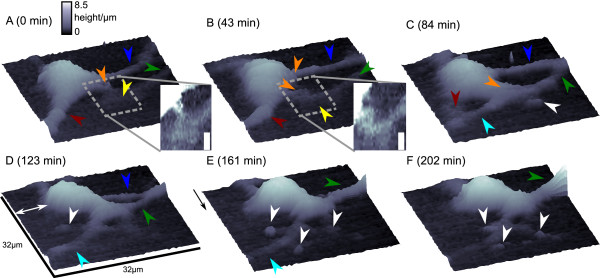
**Repeated observation of a cell from a mixed neural-glial culture**. Six scans of a cultured neural cell performed within 202 min. (A)-(F) Three dimensional plots of the data. Scan dimensions are indicated in (D), height indicated by the color gradient at the top of frame (A), main scanning direction is indicated by the arrow at the upper left corner in (E). Insets in (A) and (B) display a magnification of the area marked by the dashes with increased contrast, white scale bar indicates 3.5 μm. Arrows mark different cell parts that changed during the time of observation. Red: Old main process of the observed cell, orange: new leading process, blue and green: upper and lower process of the 2^nd^ cell, respectively, yellow: terminal fan-formed structure of the process marked green, cyan: novel process, white: newly emerging membrane protrusions. White double-headed arrow in (D) marks a change in position of the cell body. Lateral step size was 500 nm, vertical step size was 100 nm, frame acquisition time was about 30 min.

Figure 3 shows three dimensional plots of the data interpolated by cubic splines of six successive scans of a cultured neural cell obtained within 202 min. Lateral step size was 500 nm, vertical step size was 100 nm, acquisition time was about 30 min per frame. A movie composed of the top views of the data is available [see Additional File 2]. The red arrow in Fig. 3A marks the most prominent process of the observed cell, the blue and green arrows mark two processes most likely originating from a cell outside the scan area. The yellow arrow marks a fan formed structure at the terminal part of the process marked by the green arrow. The structure becomes more apparent in the magnification with increased contrast (inset). The orange arrow marks a small cell extension speculatively in contact with the previously mentioned fan-formed structure. During the 43 min interval between the first two scans (Fig. 3B) the position of the extension putatively forming the connection between the observed cell and the cell located outside the scan area in the first image (marked by the orange arrow) has either moved towards the upper process (blue arrow) as indicated by the upper orange arrow in Fig. 3B or shifted downwards (lower orange arrow). The fan-formed structure (yellow arrow) and the lower cell extension marked by the lower orange arrow established a new contact as visible in the magnification with higher contrast (inset). The processes marked by the blue and green arrow seem to converge in a branch that becomes visible in the upper right of the image. Also note the change in shape of the prominent cell process (red arrow).

Further 41 min later (Fig. 3C) the cell shape had changed considerably. The major process of the observed cell (marked by the red arrow) underwent a severe reduction in size whereas the protrusion forming the putative cell-cell contact had grown towards its target (orange arrow). The former fan-shaped sturcture is no longer visible. The detailed structure of the most likely overlapping membranes remained unresolved. However, a novel membrane protrusion originated from this structure (white arrow) and a novel process developed (cyan arrow). One might also interpret this process as the old major process marked by the red arrow in the previous images. At the same time the putative target cell had moved closer towards the cell in the scanning frame such that the root of the two branches (blue and green arrows) now was located in the scan area. In the fourth scan obtained (Fig. 3D) the position of the cell body had changed (white double headed arrow) and the root of the branch formed by the two processes (marked green and blue) had further shifted into the scan area. Note the novel cell protrusion marked by the white arrow and that the upper process of the branch (marked blue) had become less prominent.

At *t *= 161 min and 202 min the formerly large process (marked blue in the previous images) entirely vanished and a leading single process had established (green arrow). White arrows indicate unambigiously new membrane extensions indicating that the present scanning conditions do not impede process outgrowth. The structures resemble the filopodia of the neurite shown in Fig. 1 and thus could indicate that the ingrowing cell might have been a neuron.

This observation clearly demonstrates that SICM in the present configuration is able to observe spontaneously developing cell rearrangements. Because of the complex rearrangement that occured, the observed changes in the shape of cultured neural cells are most likely not induced by contacts between scanning probe and cell membrane. Both cells move in opposite directions and many membrane protrusions occur in a non-systematic manner. This supports the interpretation that the observed displacement of the neurite shown in Fig. 1 is not artificial due to probe-cell interactions as observed in SICM measurements using slightly different configurations [15].

### Observation of a migrating oligodendrocyte precursor cell

**Figure 4:**
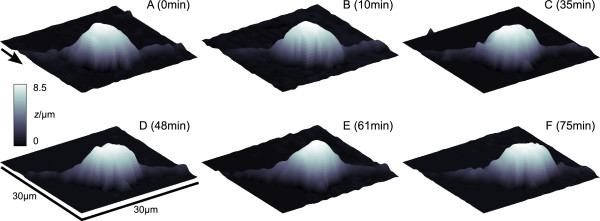
**Repeated observation of an oligodendrocyte precursor cell**. Six scans of an oligodendrocyte precursor cell recorded within 75 min. (A)-(F) Three-dimensional plots of the data. Scan dimensions are indicated in (D), main scanning direction is indicated by the arrow in (A). Note movement of the cell body into *x*- and *y*-direction (right hand side of the images). Step sizes were 1 μm in lateral and 100 nm in vertical direction.

Six successive images from a rat oligodendrocyte precursor cell were obtained within 75 minutes using a lateral step size of 1 μm and a vertical step size of 100 nm [see Additional File 3]. Acquisition time was about 10 minutes per frame. The basal plane was noise filtered using a threshold filter setting every *z*-value below 1 μm to zero. Data is shown interpolated by cubic splines in Fig. 4.

The entire cell body moved towards the *x*- and *y*-direction (right hand side of the images) during the time of observation. Clearly visible are the deformations of the cell shape, particularly of the upper part of the cell body occuring during migration. Whereas the cell height in scans A (8.3 μm), B (8.2 μm), D (8.2 μm) and F (8.2 μm) remained nearly constant it was considerably flatter in scan C (8.0 μm) and higher in scan E (8.5 μm).

**Figure 5:**
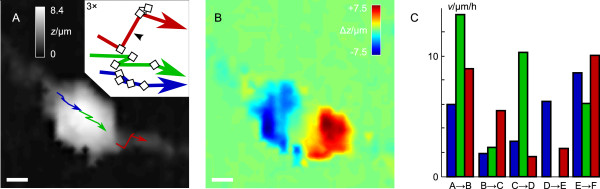
**Migration analysis of the OPC depicted in Fig 4**. (A) Trajectories of the origin of the frontal process (red), the maximum cell height (green) and the origin of the rear process (blue) superimposed on the top-view of the data of the first scan (Fig. 4 A); inset shows the magnified trajectories (rearranged for clarity). (B) Difference image between first and last scan. Lateral scale bars in (A) and (B) represent 3 μm. (C) Plots of the velocities of each of the three points of observation as defined in (A).

Fig. 5 analyzes the locomotion of the OPC in more detail. The trajectories of the origin of the rear process (blue arrow), the cell maximum (green arrow) and and the origin of the front process (red arrow) are superimposed on the top view of the data of the first scan in Fig. 5A. The frontal point was defined as the point of steepest slope in the direction of movement and the rear end as the point of steepest slope in the direction of retraction. The inset compares the trajectories (magnified three times; rearranged for clarity). Whereas between scans A and B as well as between scans C and F (Fig. 4) the cell moves into its heading direction it undergoes a change in shape leading to a lateral movement of the cell front (indicated by the red trajectory of the origin of the frontal process) between scans B and C (indicated by the black arrow-head in the inset). In contrast, the rear process only shows minor lateral movements as indicated by the trajectory of the corresponding point of observation (blue trajectory).

Fig. 5B shows the difference composed of the subtraction of the data of scan A from scan E visualizing the overall topographical changes in position during the time of observation. Again, red areas denote areas of increasing height and thus novel cell locations whereas blue areas represent areas of decreasing height corresponding to previous cell locations. Overall locomotion distances amount to 4.3 μm, 5.0 μm and 4.4 μm for frontal, maximal and rear observation point, respectively, yielding velocities of 3.4 μm/h, 4.0 μm/h and 3.5 μm/h for the respective parts of the cell.

The single average velocities of the points of observation are plotted in Fig. 5C (colors correspond to the trajectories shown in Fig. 5A). Average velocities were calculated from the locomotion distance and the time interval between two successive scans. Between frames A and B all three points of observation moved relatively fast with a velocity of about 9 μm/h (front), 12 μm/h (maximum) and 6 μm/h (rear). Hence, the point of maximal cell height nearly moved twice as fast as the rear part. Between scans B and C movement was slower. Nevertheless, the frontal point of observation moved approximately three times faster than the top of the cell and the rear part (about 6 μm/h compared with about 2 μm/h). Subsequently, the frontal and rear section nearly maintained their position while the cell maximum moved rapidly (respective velocities from back to front: 3 μm/h, 10 μm/h, 2 μm/h), followed by a distinct movement of the rear part of the cell while the highest point remained stationary. Between the last two scans all three cell parts moved rapidly yet the movements of the outer parts dominated. Most interestingly, the front end of this particular cell exhibited the largest, more exploratory movements whereas the other parts followed with smaller lateral displacements.

The determined velocities match the velocities previously determined in a detailed study of OPC migration [18] that yielded a mean migration velocity of about 10 μm/h ± 7 μm/h for OPCs on poly-L-Lysin.

During cellular activity local concentration changes at the cell surfaces may occur which can amount to approximately 10% under conditions such as enhanced neuronal activity [19]. This would affect height detection with the SICM by 10% since the resistance depends linearly on the conductance. At the surface of isolated cells in culture the equilibration of local concentration changes is expected to be speeded by membrane movements caused by water fluxes through aquaporins as well as the large diffusion space of the bath solution.

The speed with which local concentration changes are equilibrated was estimated as follows: The conductance of the bath medium is mainly carried by NaCl at the concentration *c*_0_. The diffusion coefficient of NaCl in water at room temperature is assumed as *D *≈ 10^-9^m^2^/s [20,21] and further assumed to be independent from the NaCl concentration. The distance between probe and sample that caused the observed resistance to increase to the stop criterion was approximated from approach curves and estimated to be about 400 nm. Thus potential electrolyte changes exceeding 400 nm could disturb height detection notably. As maximal possible concentration change that could distort our measurements we assume a cube with an edge length of *l *= 1 μm depleted of NaCl and located at the border of the sample surface. We define the coordinate *x *= 0 as the location of the interface between the cube, that itself is located at *x *> 0, and the bath, located at *x *< 0, in one dimension. Diffusion into the cube is described by Fick's second law, (∂*c*/∂*t*)_*x *_= *D*∂^2^*c*/∂*x*^2^ (equation 1), and the average concentration  inside the cube at time *t *is given by  (equation 2) where *c*(*x*, *t*) is the concentration at location *x *inside the cube at time *t*. Fick's second law is solved assuming the following boundary conditions: At *t *= 0 s the concentration inside the cube is *c *= 0 mM, outside the cube it is *c *= *c*_0 _and for *t *> 0 s, the concentration outside the cube is still *c *= *c*_0 _for locations far away from the cube (*x *→ - ∞). To solve the differential equation (eq. 1) we further assume *c *= 0 mM for *x *→ ∞.

Inserting the solution of eq. 1 obtained by using these boundary conditions into eq. 2 yields . Simplifying this by approximating  (note that s^1/2 ^denotes the square root of a second) results in . This yields (*t *= 0.001 s) = *c*_0_, hence, the putative concentration difference would be compensated in roughly 10 ms.

This estimation neglects the fact that ion influxes into the cube from five directions occur (assuming the sixth direction is the probe surface) and particularly the turbulences caused by the motion of the scanning tip that most likely further enhance the speed of concentration equilibration.

Since the frame aqcuisition time was about 10 minutes for scans consisting of 900 pixels and about 20 minutes for scans consisting of 3600 pixels, on the average a pixel was detected every 500 ms to 600 ms. Since this is 50 fold to 60 fold the time we estimated for the equilibration of the maximal possible concentration difference we assumed that influences in height detection due to ionic fluxes across the cell membrane were negligible. Nevertheless, they might affect SICM measurements operating in faster scanning modes.

Our records demonstrate that SICM in floating backstep operation is a suitable tool for long term recordings of single living cells in culture. Our present observations of a neurite and the rearrangements of neural processes show that the SICM can be stably operated to allow investigations on vital cell structures for more than 8 hours. Whereas SICM not yet achieves the lateral resolution of AFM measurements a lateral step size of 500 nm is already sufficient to grossly locate structures like lamellipodia. Further refinements of operation mode, software and scanning tips allow more detailed and high-speed scans of selected structures [13]. However, smaller probe tips required for higher resolution imaging detect the surface at a smaller distance between probe and sample [22] and thus might cause distortions of the cell movements [15]. Most remarkably, the ability to image entire cell somata repeatedly and to determine velocities of subcellular parts of a cell provides new options for the investigation of changes in cellular shape during migration, potentially providing a tool to investigate the subcellular distribution of activity of ion- and water channels involved in cell migration [23,24] combined with the corresponding subcellular cell surface changes.

Our results confirm that OPCs migrate in a saltatory manner [18] and indicate that the displacement of the nucleus, that presumably corresponds to the maximal *z*-value [3], and the movement of the cell soma boundaries occur in a distinct but concerted manner. Further investigation of migrating cells with backstep SICM may unravel the local dynamics during cell migration and thus help to complete our understanding of the mechanisms driving cell migration.

## Competing interests

The authors declare that they have no competing interests.

## Authors' contributions

Both authors designed the project and prepared the manuscript. PH carried out most of the SICM measurements and data analysis.

## Supplementary Material

Additional file 1 **Long term observation of the terminal part of a nerve cell process**. A movie composed of top views of the data presented in Figure 1. Click here for file

Additional file 2 **Long term observation of two neural cells**. A movie composed of top views of the data presented in Figure 3. Click here for file

Additional file 3 **Observation of a migrating oligendrocyte precursor cell**. A movie composed of the data presented in Figure 4. Click here for file
